# Oriented artificial niche provides physical-biochemical stimulations for rapid nerve regeneration

**DOI:** 10.1016/j.mtbio.2023.100736

**Published:** 2023-07-20

**Authors:** Minhong Tan, Weizhong Xu, Ge Yan, Yang Xu, Qiyao Xiao, Aiping Liu, Lihua Peng

**Affiliations:** a College of Pharmaceutical Sciences, Zhejiang University, Hangzhou, 310058, PR China; b State Key Laboratory of Quality Research in Chinese Medicine, Macau University of Science and Technology, Macau, PR China; c Key Laboratory of Optical Field Manipulation of Zhejiang Province, Zhejiang Sci-Tech University, PR China; d College of Materials Science and Engineering, Zhejiang University, Hangzhou, 310027, PR China; e Jinhua Institute of Zhejiang University, Jinhua 321299, Zhejiang, PR China

**Keywords:** Oriented artificial niche, In situ nerve regeneration, Mesenchymal stem cells, Neural differentiation, Physical-biochemical factors

## Abstract

Skin wound is always accompanied with nerve damage, leading to significant sensory function loss. Currently, the functional matrix material based stem cell transplantation and in situ nerve regeneration are thought to be effective strategies, of which, how to recruit stem cells, retard senescence, and promote neural differentiation has been obstacle to be overcome. However, the therapeutic efficiency of the reported systems has yet to be improved and side effect reduced. Herein, a conduit matrix with three-dimensional ordered porous structures, regular porosity, appropriate mechanical strength, and conductive features was prepared by orienting the freezing technique, which was further filled with neural-directing exosomes to form a neural-stimulating matrix for providing hybrid physical-biochemical stimulations. This neural-stimulating matrix was then compacted with methacrylate gelatin (GelMA) hydrogel thin coat that loaded with chemokines and anti-senescence drugs, forming a multi-functional artificial niche (termed as GCr-CSL) that promotes MSCs recruitment, anti-senescence, and neural differentiation. GCr-CSL was shown to rapidly enhances in situ nerve regeneration in skin wound therapy, and with great potential in promoting sensory function recovery. This study demonstrates proof-of-concept in building a biomimetic niche to organize endogenous MSCs recruitment, differentiation, and functionalization for fast neurological and sensory recovery.

## Highlights

• An all-in-one patch as oriented artificial niche supplies heterogeneous signal.
• Programmed delivery of physical-biochemical stimulations for accelerating in situ nerve regeneration.
• 3D nerve conduit matrix with regular porosity for enhanced nerve regeneration.
• A proof-of-concept for integrated therapy of regenerative medicine.

## Graphical abstract

The oriented patch as artificial niche provides physical-biochemical hybrid stimulations for rapid nerve regeneration.

**Figure:**
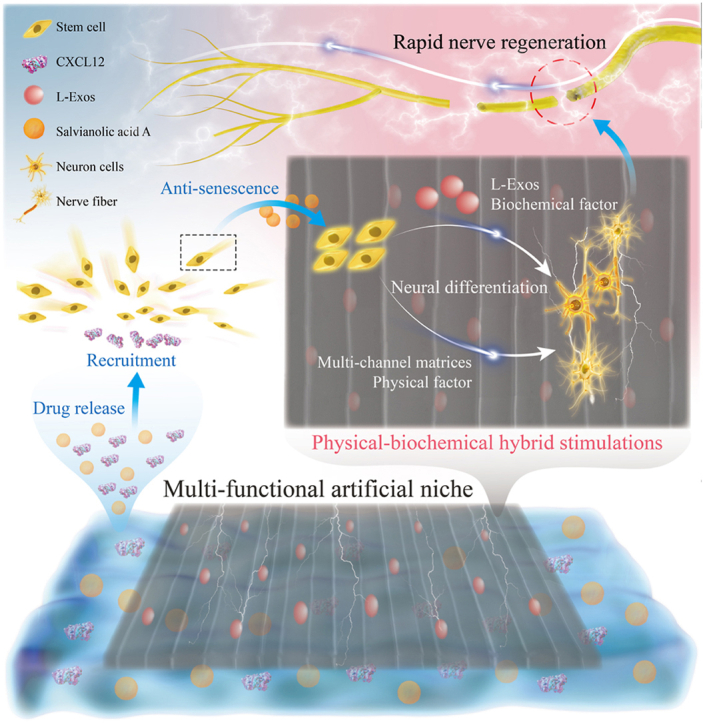

## 1 Introduction

In the treatment of nerve injury, the recovery of cutaneous nerve excitation function and the regeneration of neural network is often neglected in the healing process of various skin wounds, resulting in innervation dysfunction, decreased patient quality of life, and increased socioeconomic burden. The current neural/stem cell transplantation-based treatments face many challenges, such as poor cell survival rate, limited differentiation, ineffective engraftment, immune resistance, and lack of excitation functions [1,2]. Recently, endogenous mesenchymal stem cell (MSCs)-based in situ nerve regeneration, which avoids the drawbacks of cell transplantation, has attracted increased attention [1,3]. Generally, in situ nerve regeneration includes the recruitment, proliferation, and neural differentiation of MSCs and is affected by multiple physical and biochemical cues. However, in most pathological conditions, these signals are absent, leading to difficulties in nerve regeneration. Therefore, an efficient therapeutic system that transmits both biophysical and biochemical signals appear promising.

Matrices with aligned porosity and linear features on the micrometer scale are useful for guiding nerve fibers across multiple length scales [4]. Surface-contacting cells, with a balance between hydrophilicity and hydrophobicity, also affect adhesion and growth [5]. In addition, good molding ability and adequate mechanical strength are required to retain space for cell incorporation and growth [6,7].In addition, because nerve cells are electrically active, modulating electrical signals using conductive functional materials to promote neural differentiation might be favorable [8]. Thus, the use of biomaterials to satisfy the above biophysical stimulation is critical. Currently, chitosan is widely used in tissue engineering owing to its inherent advantages such as hydrophilicity, biodegradability, and biocompatibility [9]. However, the mechanical properties of chitosan are generally poor, which limits its further application [10]. To address this problem, graphene derivatives, graphene oxide (GO), and reduced graphene oxide (rGO) with a high specific surface area, mechanical strength, and conductivity can be co-manufactured to remedy the deficiency and improve performance [11]. Therefore, a self-assembled matrix based on chitosan and GO or rGO crosslinking could be a novel technique to coordinate the mechanical properties, amphipathy, and conductivity of materials for tissue engineering. In this study, oriented freezing technology, also known as freeze casting technology, was utilized to prepare chitosan- and GO- or rGO-based 3D multichannel matrices with guiding and orienting channels as a simple, efficient, environmentally friendly, and controllable method of material structure customization. The pore size and mechanical strength of the matrices can be precisely adjusted by adjusting the processing parameters, and a conductive cell-interaction interface for charge transfer can be created to enhance the bioelectricity signal and promote the transmission of nerve signals.

Additionally, nerve regeneration is regulated by several biochemical factors. Chemoattractants have shown great potential in activating the migration of endogenous MSCs from the surrounding tissue and bone marrow towards injury sites for the recruitment of stem cells [12]. Among these, Chemokine C-X-C motif chemokine ligand 12, CXCL12, one of the most thoroughly investigated chemoattractants for stem cell recruitment, plays a pivotal role in the mobilization and homing of MSCs as the seeding cells for the following regeneration [13,14]. It is worth mentioning that MSCs proliferation is accompanied by the accumulation of cellular damage and alterations in intrinsic cell signaling pathways, which ultimately leads to the dysregulation of proliferation and differentiation, replicative senescence, loss of stemness, and consequent damage to regenerative capacities [15]. Small chemical molecules and growth factors, such as fibroblast and hepatocyte growth factors [[16], [17], [18]], [[16], [17], [18]] [[16], [17], [18]] are normally used to maintain the stemness of stem cells. However, screening for effective chemical molecules is time-consuming, because growth factors are expensive, easily degrade, and have limited effects. Hence, tactics that take advantage of naturally active molecules to tackle the aforementioned issues to boost cell proliferative capacity and diminish replicative senescence of MSCs have been proposed to enhance the success rate of stem cell-based therapies. In our previous study, Salvianolic acid A (SA) was identified for its excellent efficacy in retarding the senescence of MSCs by decreasing SA-β-gal levels and protecting telomerase activity, providing a novel natural agent with high efficiency and low toxicity to cells for anti-senescence.

Because of their high molecular weight, hydrophilicity, and negative surface charge, the most often used biofactors, growth factors, are always challenged with poor bioavailability, low stability, and limited efficacy in directed neuronal development [19,20]. Exosome-like vesicles (ELVs) derived from medicinal plants are promising therapeutics [21]. Because of their stable structure, nanoscale size, and intrinsically expressed transmembrane and membrane-anchored proteins, ELVs are good vehicles to facilitate the cellular uptake of incorporated RNAs in target cells to enhance their therapeutic effects [[22], [23], [24]]. As a frequently used medicinal plant for neuroprotection [25,26], in this study, for the first time, we isolated ELVs from Ligusticum chuanxiong hort (L-Exos) and demonstrated that L-Exos has great potential to stimulate the nerve regeneration of MSCs.

**Fig. 1:**
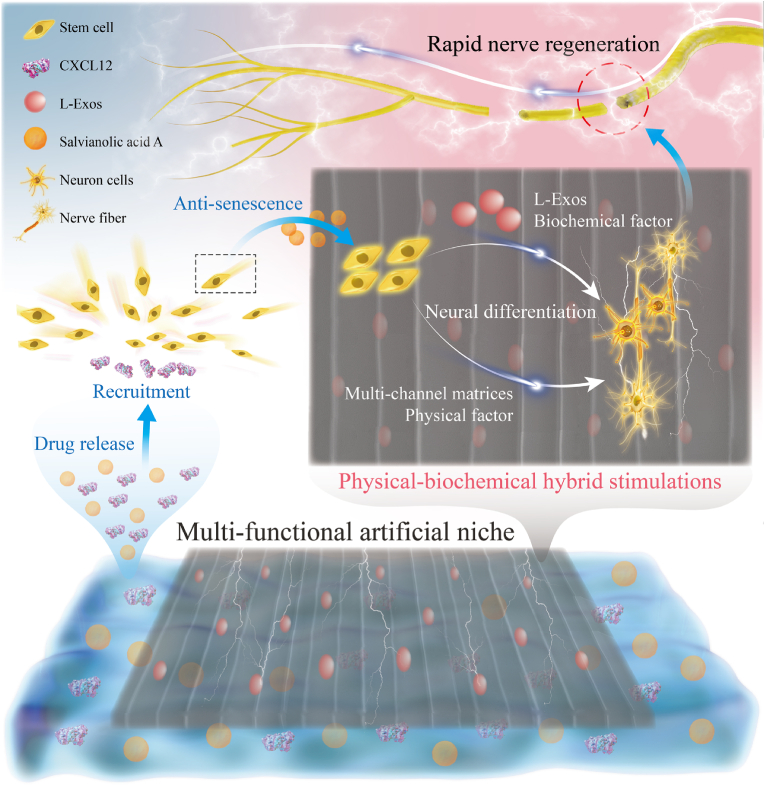
**Scheme of the structure and therapeutic procedures of GCr-CSL oriented patch for in situ rapid nerve regeneration.** The GCr-CSL oriented patch acts as a multi-functional artificial niche, accelerating rapid nerve regeneration through the following major steps: I) CXCL12 released from the hydrogel layer of the GCr-CSL patch recruits MSCs from the bone marrow or surrounding tissue to the injury site; II) Salvianolic acid A released from the same hydrogel reservoir inhibits the senescence of recruited MSCs, which also promotes their proliferation; and III) L-Exos incorporated in the matrix combined with the microenvironment of 3D multi-channels promote neural differentiation and nerve regeneration of the recruited MSCs by providing physical-biochemical hybrid stimulations; thus, wound healing accompanied by the regeneration of skin nerves.

Herein, based on the above information, we propose a novel multi-functional patch as an artificial niche (L-Exos-loaded CS-rGO that was compacted by GelMA-coated CXCL12, SA), termed the GCr-CSL patch, that stimulates the recruitment, anti-senescence, and integrates biophysical and biochemical signals to promote nerve regeneration based on endogenous stem cells (Fig. 1). GCr-CSL is assembled by a methacrylate gelatin (GelMA) hydrogel coat for CXCL12 and SA co-releasing, and a chitosan-rGO composite matrix containing L-Exos for hybrid neural-directing stimulating. GCr-CSL not only releases various biological factors in a controlled manner but also provides a matrix with optimized physical stimulating factors, including regular and uniform porous structure, suitable mechanical strength, and conductivity to synergistically stimulate skin nerve regeneration. This study demonstrates a proof-to-concept in organizing chemokine, inhibiting senescence molecules, and neural directing physical-biochemical heterogeneous cues within an oriented artificial niche for accelerating nerve regeneration, possessing great potential in skin neural repair applications.

## 2 Materials and methods

### 2.1 Fabrication of 3D nerve matrices

The Chitosan (CS, Degree of deacetylation ≥95%, Viscosity 100–200 MPa s, Aladdin) solution (40 mg/mL) was prepared by dissolving chitosan powders in aqueous solution with 2% acetic acid for 24 h. The graphene oxide (GO) nanosheets were prepared by oxidizing natural graphite powders via a modified Hummers’ method reported [27]. GO suspensions (2 mg/mL) were prepared by dispersing in deionized water with ultrasonication (500 W, JY92-IID) for 30 min And rGO (reduced graphene oxide, 2 mg/mL) suspensions were obtained by reducing GO suspensions with sodium ascorbate (4 mg/mL) at 90 °C for 4 h, where lauryl sodium sulfate (8 mg/mL) was used as a surfactant. The CS-GO and CS-rGO solutions were obtained by dissolving chitosan in these suspensions. Before freezing, all the solutions were vacuumed to remove air bubbles and cooled to 4 °C.

3D nerve matrices were fabricated by the ice template method [28], The matrices with directional porous structures (CS-D, CS-GO-D, and CS-rGO-D) were prepared by directional freezing, typically, the solutions were poured into molds and placed on the surface of a copper cold finger (−90 °C) with a bottom-up directional temperature gradient. For random matrices (CS-R), the CS solution was placed at −90 °C without temperature gradient. After the solution was frozen entirely, the samples were freeze-dried for more than 48 h at −80 °C with a freeze-dryer. Afterward, the pre-matrices were then soaked in a 95% ethanol solution in 0.4% NaOH for 30 min, rinse with water, and then cross-link in 1% genipin solution (Baoji Herbest Bio-Tech Co.,Ltd) for 48 h. Finally, the 3D nerve matrices were lyophilized again and stored at room temperature.

### 2.2 Characterizations of 3D nerve matrices

(i) SEM images were gathered by ULTRA 55-36-73 at an acceleration voltage of 10 kV [29].
(ii) *Porosity test.* The porosity of the nerve matrices was measured by an ethanol replacement method. The porosity was calculated using the following equation:

$$Porosity\left(\%\right)=\frac{\left(M_{1}-M_{0}\right)\times \rho }{V}V\times 100\%$$

where *M*_*1*_ was the mass of the samples after immersion in ethanol for 24 h; *M*_*0*_ was the initial mass of the sample; *V* was the volume of the samples and *ρ* was the density of ethanol (*ρ* = 0.789 g/cm^3^).

(iii) *Mass change ratio test.* The matrices were dried at 80 °C for overnight, weighed and immersed in PBS (37 °C, 5% CO_2_). At the different time points, the matrices were taken out and the amount of water absorbed was determined by weighing. The mass change ratio of the sample was calculated using the following equation:

$$Mass\;change\;rate\left(\%\right)=\frac{W_{S}-W_{d}}{W_{d}}\times 100\%$$

where *W*_*s*_ and *W*_*d*_ are swollen weight and dry weight of the sample, respectively.

(iv) *Degradation test.* In short, rinse with deionized water under simulated physiological conditions, then dry and weigh, and estimate the percentage of degradation by dividing the weight loss by the initial dry weight.

$$Degradation\left(\%\right)=\frac{W_{0}-W_{t}}{W_{0}}\times 100\%$$

where *W*_*t*_ and *W*_*0*_ are the dry mass of the sample after immersion in PBS and the initial mass of the sample.

(v) *Mechanical test*: Mechanical tests were conducted under conditions of relatively few times at high strain according to previous reports [30,31]. The compressive properties of nerve matrices were determined using a universal tester (Instron 2456) equipped with two flat-surface compression stages and 5 N load cells. The samples were compressed in a direction perpendicular to the temperature gradient direction. All stress-strain curves were obtained at the displacement rate of 10 mm/min and a strain of 60%. Prior to mechanical testing, samples drying at 80 °C for overnight, while wet samples were immersed in PBS for overnight to reach swelling equilibrium. All mechanical testing was carried out at least three samples and the average value was reported.

### 2.3 Functional evaluation of 3D nerve matrices in vitro

(i) *Isolation and culture of PC12:* PC12 cell line was obtained from the cell bank of Shanghai Institute of Biological Sciences, Chinese Academy of Sciences. PC12 cells were cultured in DMEM supplemented with 10% FBS and 1% double antibiotics. The cells were cultured in a sterile cell incubator with a culture environment of 37 °C, 5% CO2, and 100% humidity. The medium was changed every 2 days. After the cells in the culture bottle reached 80–90%, the cells were isolated by 0.25% trypsin/EDTA solution for passage operation. Before cell inoculation, the dried nerve scaffold was placed in a glass culture dish, autoclaved at 120 °C for 30 min, then the sample was transferred to a 24-well plate, sterilized in 75% ethanol for 2 h, washed three times with PBS, and irradiated with ultraviolet light for 1 day. Finally, the scaffold was immersed in cell culture medium overnight to ensure that the scaffold was completely hydrated by cell culture medium. Subsequently, PC12 cells were inoculated on the scaffold with a cell density of 2 × 10^5^/mL, and the scaffold inoculated with cells was cultured in the incubator for 1,3,5,7 and 9 days, and the medium was changed every two days.
(ii) *Cytotoxicity and proliferation tests*: Leaching solution method was used to evaluate the cytotoxicity of PC12 cells. First, the sterilized sample was immersed in complete medium for 72 h to obtain a reliable leaching solution. Then, PC12 cells were cultured in 96-well plates at a cell density of 5 × 10^3^/100 μL, and the cell culture medium was added. The cells were cultured in the cell incubator for 12 h, and the medium was replaced with the leaching solution (100 μL) on the first and second days after the medium change. PC12 cells were seeded in a neural scaffold (side length 3 mm × 3 mm, thickness 1 mm) at a cell density of 200/100 μL and cultured in 96-well plates. CCK-8 solution (10 μL 10 vt %) was added to each well and incubated in an incubator for 4 h. The absorbance at 450 nm was measured using a microplate reader, and the data obtained from a 96-well plate containing a known number of living cells were used as the calibration curve. The kits for calcein acetyloxymethyl (calcein AM) and ethidium homodimer dyes were used to determine live and dead cells, respectively. PC12 cells were cultured in 96-well plates at a cell density of 5 × 10^3^/100 μL per well. Calcein AM and Ethidium homodimer dyes were added and cultured in a cell incubator for 15 min. Then, live cell imaging was performed using CLSM, and live cells (green fluorescence) and dead cells (red fluorescence) were counted using Image J software. Cell viability and cell proliferation rates are calculated using the following formula:

$$Cell\;Viability\left(\%\right)=\frac{{OD}_{e}-{OD}_{b}}{{OD}_{c}-{OD}_{b}}\times 100\%$$

$$Cell\;Proliferation\;rate={OD}_{e}-{OD}_{b}$$

OD_e_, OD_b_ and OD_c_ were the average OD values of the experimental group, the blank control group and the control group, respectively.

(iii) *Confocal laser scanning microscope Imageing:* The nerve scaffolds inoculated with PC12 cells for 1,3,5,7,9 days were fixed in 4% formaldehyde and placed at 4 °C for 12 h. Next, the PBS solution was rinsed and gently blown, washed 3 times, 5 min each time, and then permeabilized in 0.5% Triton-X-100 solution for 1 h. After that, the samples were rinsed with PBS, and about 50 μL of rhodamine-labeled phalloidin (200 nM) was added to the surface of the material at room temperature for staining for 2 h. Then, PBS was rinsed and 0.5 mL DAPI (10 μg/mL) was added for staining for 1 h under dark conditions. Image J software was used to record the number of cells per unit area, average neurite length and percentage of neurite cells in PC12 cells. From the starting point of PC12 cells to the tip of the neurite, the angle of neurite orientation relative to the scaffold channel was measured and 0° represents the direction of the channel.
(iv) *Calcium imaging.* For electrical stimulation, 2.5 μmol of Fluo-4-AM (Beyotime) dye was added to the CS-rGO-D scaffold inoculated with cells, incubate in the dark for 30 min at 37 °C and 5% CO_2_, then remove the material, and use copper wire and silver glue. A wire was connected to both sides of the stent, and a series of single-phase cathodic pulses of ∼100 ms were applied on both sides of the stent, with an interval of 20 s, and the stimulation threshold was 0.1 V. CLSM images the time-lapse calcium level of PC12 living cells, and FV100 software is used to measure the fluorescence intensity of each cell.

### 2.4 Isolation and characterizations of L-Exos

(i) *Isolation of L-Exos*: For isolation and purification of exosomes, L-Exos were isolated from Ligusticum chuanxiong hort juice (L-Exo, The Ligusticum chuanxiong Hort Base of Western Sichuan Plain, Sichuan Province, China) by differential centrifugation. Briefly, Ligusticum chuanxiong hort was ground in a blender to obtain juice, then the juice was centrifuged first at 3000 g for 30 min and then at 10,000 g for 1 h to remove large fibers. The supernatant was ultracentrifuged at 150,000 g for 2 h, and the pellet was suspended in PBS. Then, the suspension was ultracentrifuged again at 150,000 g for 2 h to obtain pure L-Exos. The quantified exosomes were stored at −80 °C until use.
(ii) *Characterizations of L-Exos*: The concentrations of L-Exos obtained were quantified based on protein concentration using BCA Protein Assay (Boster biological technology). L-Exos were characterized with respect to particle sizes, and surface charge (represented by the surface zeta potential) was measured using laser diffraction spectrometry (Malvern Zeta sizer 3000HS, Malvern) [24]. After screening of size and concentration, L-Exos were prepared for transmission electron microscopy (TEM) imaging [32], a drop of the sample was deposited onto the surface of a formvar-coated copper grid, after which 1% uranyl acetate was added for 15 s and the sample was allowed to dry at room temperature for subsequent imaging.

### 2.5 Construction and characterizations of GCr-CSL

(i) *Construction of GCr-CSL patch.* One slice of 3D nerve scaffold (CS-rGO-D) loaded with L-Exos was put into a polydimethylsiloxane square mold (20 mm × 20 mm × 1.5 mm), then the pre-GelMA solution (EFL-GM-60, 60% graft degree) and lithium phenyl-2,4,6-trimethybenzoylphosphinate mixed with CXCL12 (Peprotech) and Salvianolic acid A (SA) was added to the mold and spin coating the scaffold, then the whole mold was exposed to 6.9 mW/cm^2^ UV light (360–480 nm) for 60 s to form the GCr-CSL patch.
(ii) *Degradation test.* GCr-CSL were placed in 2 mL of 0.5 U/mL collagenase type II solution (Solarbio Life Science) and incubated in a shaker at 37 °C, 100 rpm. At each specific time, the remaining samples were weighed after being washed with deionized water and lyophilized. The degradation rate of the patch was evaluated by calculating the weight difference between dried samples and untreated samples.
(iii) *Mechanical test.* Tensile tests of the patch were measured on a TA Instruments (DMA Q800) operating in the tension mode. Samples were fixed by two clamps of mechanical tester. The samples were stretched at a constant rate of 5 mm/min at room temperature. The strain and stress were derived from the displacement and load data respectively by normalizing to sample length, width, and thickness.
(iv) *Drug release test.* Release of CXCL12, SA, and L-Exos from the GCr-CSL: As described above, CXCL12, SA, and L-Exos were respectively loaded in Gel and CS-rGO-D scaffold to form the whole patch, three detection methods were used to determine the release behavior of patch. Briefly, 1 g of Gel contained 500 ng CXCL12 or 2 mg SA, one piece of CS-rGO contained 1.5 mg L-Exos constitute the drug release patch, which was placed into a dialysis bag, then placed in the centrifugal tubes containing 25 mL of the release medium, which were placed on a shaking box at 37 °C and 100 rpm. At pre-scheduled time intervals, 1 mL of the solution was taken and supplemented with the fresh PBS of the same volume. The CXCL12 concentration in the collected sample was assayed using a CXCL12 Elisa kit (Jiangsu Meimian Industrial Co., Ltd). The SA concentration was assayed by UV (285 nm). The L-Exos concentration was assayed by using a microBCA assay (Boster Biotech).

### 2.6 Functional evaluation and biocompatibility of GCr-CSL patch or 3D nerve matrices in vitro

(i) *Isolation and culture of BMSCs.* The isolation and culture of BMSCs were performed as previously described [33]. Sprague-Dawley rats are used in this experiment, and the test procedures are in accordance with Zhejiang University Laboratory Animal Welfare Guidelines. Briefly, rat femurs were excised from the epiphysis, and bone marrow was flushed out using a syringe with Dulbecco's Modified Eagle Medium (Gibco BRL) supplemented with 10% fetal bovine serum (Gibco BRL), l-glutamine, penicillin (50 U/mL), and streptomycin (50 U/mL). The cell suspension was placed into a 75 cm^2^ tissue culture flask (Corning) and cultured in an incubator (HF90, Health Force) at 37 °C in 5% CO_2_. Subconfluently first passage cells were detached from the flask with 0.25% trypsin-EDTA for 1 min at 37 °C. The second to fifth generation BMSCs were employed for subsequent experiments.
(ii) *Biocompatibility of the patch to cells.* To investigate the cells' growth and adhesion on GCr-CSL, cells were rinsed with PBS after 24 h of culture on GCr-CSL. Cells were fixed with 4% paraformaldehyde solution (Solarbio Life Science) for 10 min, permeabilized with 0.1% Triton X (Sigma–Aldrich) for 15 min and blocked with 5% bovine serum albumin (Sigma–Aldrich) for 30 min. The cells were then stained with actin-tracker green (Beyotime) at a 1:200 dilution for 60 min. After that, the nuclei were stained with DAPI (Solarbio Life Science) for 10 min and imaged under a confocal fluorescence microscope (CLSM, OLYMPUS BX61).
(iv) *Cells migration assay.* The BMSCs migration was tested by the RTCA DP instrument (ACEA Biosciences Inc.) [34]. The top chamber of the modified 16-well plates was filled with serum-free medium, and the membrane is required to be hydrated and preincubated in the CO_2_ incubator at 37 °C for 1 h before obtaining a background measurement. After this incubation period, BMSCs suspension was seeded into the upper chamber applying 3 × 10^4^ cells in 100 μL. Bottom chambers contained media with GCr or GCr-C to assess migration when exposed to GCr or GCr-C. The E-Plate 16 is assembled by placing the top chamber onto the bottom chamber and snapping the two together. Then, the E-Plate 16 was placed in the RTCA DP station and the impedance value was automatically monitored every 5 min for 48 h and expressed as a cell index value. All data was recorded by the supplied RTCA software.
(iii) *Evaluation of inhibition of replicative aging of the patch to BMSCs.* [35] BMSCs cultured in a 6-well plate were treated with 500 μmol/L H_2_O_2_ for 24 h to induce senescence. For staining, first aspirate the cell culture medium and wash with PBS, then add 1 mL fixative, fix at room temperature for 15 min and aspirate it. After washing with PBS 3 times, the supernatant was removed and *β*-galactosidase staining solution was added. After overnight incubation in a CO_2_ free incubator at 37 °C, cell staining was imaged under an inverted light microscope.
(v) *Neural differentiation of the patch to BMSCs* in vitro*.* To investigate the neural differentiation, 4 × 10^4^ BMSCs were seeded onto the flexible-bottomed culture plates. L-Exos, GCr-L, or GCr-SL were added or suspended into the supernatant every 2 days, the medium was also removed and replenished with fresh medium in the meantime. After being cultured for 7 days, cells were washed with PBS, fixed in 4% paraformaldehyde for 30 min, and then permeabilized with 0.1%Triton X-100 (Sigma–Aldrich) for 20 min and blocked with 10% goat serum (Boster Biological Technology) for 30 min. The samples were then incubated overnight at 4 °C with anti-Nestin antibody (1:200, Cell Signaling) and detection was achieved by subsequent incubation with FITC conjugated goat anti-mouse IgG H&L (1:100, Boster Biological Technology) for an hour at 37 °C, followed by DAPI staining and imaged under a CLSM (OLYMPUS BX61) [36].

### 2.7 Animal experiment

(i) *Treatment:* Forty-two male Sprague Dawley rats (Shanghai SLAC Laboratory Animal Co. Ltd., China), each weighing 120–150 g. All animals were maintained under constant conditions (temperature 25 ± 1 °C), with free access to standard diet and drinking water. All animal experimental procedures were performed in obedience to the guidelines and protocols of the Animal Experimental Ethics Committee of Zhejiang University (ZJU20170733). The animal study referred to the method of a previous study that was slightly modified [37]. The animals were anesthetized with an intraperitoneal injection of 3% sodium pentobarbital (30 mg/kg). Full-thickness excision wound along with all the skin nerves removed were made symmetrically (1.5 cm × 1.5 cm) by scalpel excision on the depilated back of each rat. Rats with skin wounds were randomly divided into 6 experimental groups (n = 7 mice per group). Blank, CX-S-L, GCr, GCr-C, GCr-SL, and GCr-CSL were applied to the wound respectively. After treatments, all groups were dressed with transparent tegaderm to prevent infection and rehydrating of the wounds. The development of wound healing was recorded by taking pictures every 2 days and measuring the wound area using Image J software. And the weight of the rat was also recorded. The wound healing rate was calculated by dividing the area on day 0 minus area on day n by the area on day 0.
(ii) *Histological analysis.* [38] Histological analysis was performed for the healed skin tissues and organs 15 days after skin wound treatment. Retrieved samples were fixed in 4% buffered paraformaldehyde, dehydrated and then embedded in paraffin or OCT compound for slice preparation. The slice sections (5 μm in thick) were stained with the Masson's trichrome staining (KeygenBiotech), according to the manufacturers' protocol. The stained skin sections were observed and photographed with laser scanning confocal microscopy. Additionally, the organs were extracted, and the heart, liver, spleen, lung, and kidney were cut into smaller sections, fixed in 4% paraformaldehyde, embedded in paraffin, and sectioned into 5 μm of slices. The organ sections were stained with H&E and were visualized by laser scanning confocal microscopy for the histological study of toxicity.
(iii) *Immunofluorescent staining and Imaging.* The tissues of the wound regions were retrieved on day 2 and day 15 after the treatment and embedded in an optimal cutting temperature compound, followed by freezing and slicing into 10 μm thick sections at −22 °C. To visualize the migration and the regenerated nerves of BMSCs *in vivo*, tissue sections were stained with antibodies against CD29 and CD90, Nestin and β3-tubulin respectively. CD29, CD90, Nestin and β3-tubulin signals were visualized using corresponding FITC and Cy3-conjugated secondary antibodies respectively. The nucleus were all stained by DAPI. Images of the samples were observed under laser scanning confocal microscopy. Images were photographed from 6 random areas for the quantification of fluorescence intensity. All images were post-processed and quantified using Image J software.
(iv) *β-Galactosidase(β-Gal) Staining.* The tissues of the wound regions were retrieved on day 15 after the treatment, followed by freezing and slicing into 6 μm thick sections at −22 °C. After being fixed in 4% paraformaldehyde for 30 min and washed with PBS 3 times, β-galactosidase staining fixative was then added to fully cover the tissue. The samples were sealed with plastic film to prevent evaporation. After incubating overnight at 37 °C, the samples were washed with alcohol (70%) for 10 min and PBS 3 times and then mount with glycerol. Images of the samples were observed under a brightfield microscope.
(v) *Western blot analysis.* Samples were obtained from the rat wound area and homogenized by T-PER tissue protein extraction reagent (Thermo Pierce). The protein concentration was determined using a bicinchoninic acid protein assay (Beyotime). Western blot analysis was performed using 10% sodium dodecyl sulfate-polyacrylamide gel electrophoresis. After the proteins were transferred onto PVDF membranes (Millipore), they were probed with antibodies against Nestin (Abcam), β3-tubulin (Abcam) were incubated with corresponding secondary antibody for 1 h at room temperature. Then, the blots were developed using SuperSignal® West Dura Extended Duration Substrate (Thermo Pierce) and recorded on X-ray film (Fuji super RX) and the bands were imaged and quantified (n = 5 different samples per group) using an Image J [39].

### 2.8 Statistical analysis

Unless otherwise stated, data were expressed as mean ± standard deviation. For comparisons between two groups, means were compared using unpaired two-tailed Student's t-tests. A one-way analysis of variance with posthoc Tukey's honest significant difference was conducted for multiple sample analyses. All statistical analyses were performed using GraphPad Prism version 8 software (GraphPad Software Inc.).

## 3 Results

### 3.1 Construction and characterization of CS-rGO matrix

The size and shape of the 3D nerve matrix can be fine-tuned by changing the concentration of the solution and the freezing temperature, making it possible to create a range of complex shapes suitable for different applications [40]. Accordingly, four different types of matrices, CS-R, CS-D, CS-GO-D, and CS-rGO-D, with directionality or randomness with 3D multi-channels were fabricated by freeze casting technology, and the ice crystal growth was controlled by a flat copper plate. First, the influence of chitosan concentration and cryogenic temperature on pore size and shape was evaluated.

**Fig. 2:**
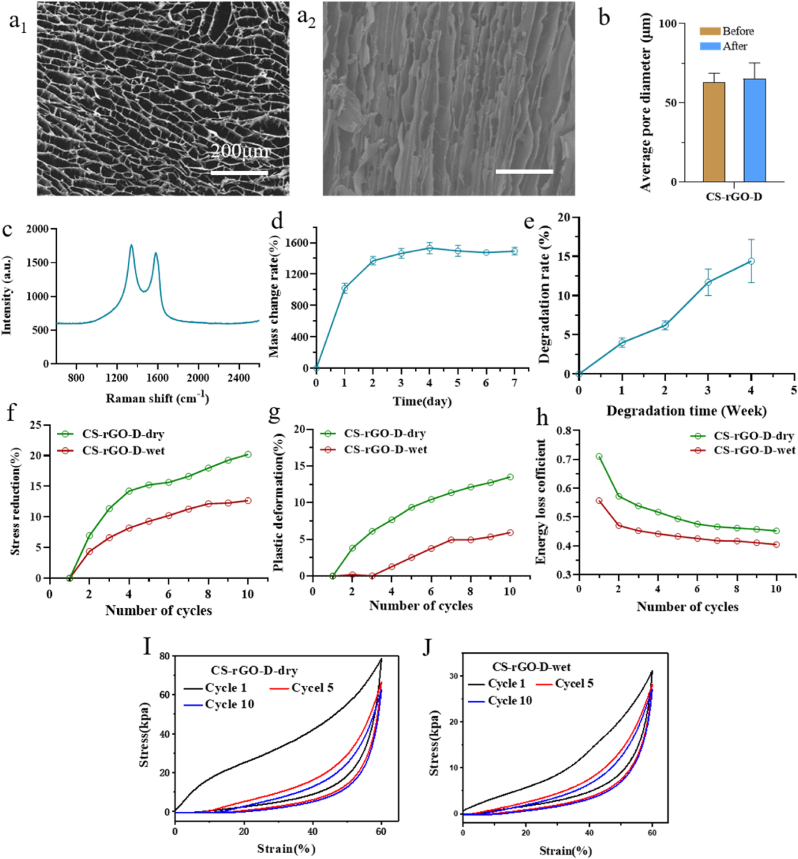
**Fabrication and characterization of the CS-rGO-D matrix.** (a_1_, a_2_) Horizontal and vertical microstructure of CS-rGO-D matrix. (b) Pore sizes of CS-rGO-D matrices. (c) Raman spectrum of CS-rGO-D matrix. (d) Mass change ratio of CS-rGO-D matrix over 7 days. (e) Degradation rate of CS-rGO-D matrix in wet environment over 4 weeks. (f–h) Stress reduction, plastic deformation, and energy loss coefficient of CS-rGO-D matrix under dry and wet environment, respectively. (I, J) Stress-strain curve of CS-rGO-D matrices under dry environment and wet environment.

The results showed that as the chitosan concentration increased, the pore structure became more regular, structural defects decreased, and the channel diameter gradually decreased with the decrease in temperature. This was mainly because as the freezing temperature decreased, the nucleation of ice crystals increased and the growth rate of ice crystals accelerated, resulting in smaller pore size and more pores (Fig. S1). To introduce conductive properties into the neural matrix, we also prepared CS-GO-D and CS-rGO-D matrices, as shown in the SEM images, the resulting CS-rGO-D matrix after lyophilization exhibited more regular multi-channel, mostly internally parallel, and fewer structural defects than the other groups (Fig. 2a_1_, a_2,_ Figs. S2a–c).

The channel diameter of the CS-rGO-D matrix (41.77 ± 5.53 μm) was smaller than that of the CS-R matrix (136.35 ± 21.64 μm) (Fig. 2b, Fig. S2d), and the radial order structure was better for pre-aligning cells, guiding cell infusion, and inducing elongation. Raman spectroscopy successfully verified the introduction of graphene-based conducting components in the neural matrix (Fig. 2c, Fig. S3a), and the CS-rGO-D matrix showed two distinct peaks at ∼ 1360 and ∼ 1590 cm^−1^ corresponding to the D band of *sp*^3^ defects of carbon atoms and the G band of graphitic carbon, respectively. The density and porosity were also determined (Table S1). As presented, the CS-rGO-D matrix showed the lowest density (70.34 ± 5.53 mg/cm^3^) and the highest porosity (92.58 ± 2.69%). In addition, the nerve matrix should have limited swelling to avoid axonal compression [41]. The mass change ratio of the CS-rGO-D matrix reached an equilibrium point at approximately 1358% after four days of soaking (Fig. 2d, Fig. S3b); nonetheless, there was no obvious damage or volume change to the matrix after seven days of soaking. An appropriate biodegradation rate for the nerve matrix is also crucial since it not only influences the mechanical properties but also needs to support and match the nerve cell growth status while not intervening with normal tissue growth during neural development [42,43]. Among the matrices, the degradation rate of the CS-rGO-D matrix reached the highest (14.4 ± 2.8%) after 4 weeks of soaking (Fig. 2e, Fig. S3c), indicating that the matrix can withstand the severe physiological environment over the *in vivo* regeneration time.

In addition, as a neural graft, it should be adjustable to complex mechanical behaviors after implantation, such as compression, stretching, torsion, and bending, which is a precondition for the stable execution of function over a long period [44]. Therefore, the matrix should have excellent resistance to deformation in dry and wet environments. To demonstrate this, compressive tests were performed at 60% strain for 10 cycles in dry and wet environments. Meanwhile, we evaluated several characteristic indexes, such as the compression modulus, stress reduction, plastic deformation, and energy loss coefficient. In detail, compared to other nerve matrices, after 10 compress-release cycles, the CS-R matrix has the lowest compression modulus (5.07 ± 5.47 and 2.16 ± 6.27 kPa), highest stress reduction (33.72% and 36.46%), plastic deformation (28.60% and 30.24%) and energy loss coefficient (0.47 and 0.47) when undergoing dry and wet environments (Fig. 2f–j, Figs. S3d–f, Fig. S4). Others, on the other hand, exhibited distinct resistance to compression and could fully restore to their original state after 10 cycles without any obvious damage. This verified that the inner directionality induced by the oriented freezing technique exerts an underlying impact on the mechanical properties of the matrix. Specifically, for the CS-rGO-D matrix, it showed 149.83 ± 2.22 and 37.28 ± 8.57 kPa compression modulus (Table S1), 20.22% and 12.67% stress reduction, 13.53% and 12.67% plastic deformation, and 0.45 and 0.40 energy loss coefficient under dry and wet environments, respectively. In conclusion, the CS-rGO-D matrix, with a uniform and ordered honeycomb multi-channel structure of suitable size, possesses the most favorable mechanical properties for application in physiological environments and provides a critical guided structure for stem cell development and nerve development.

### 3.2 CS-rGO-D matrix induces proliferation, directional growth, and axonal elongation of PC12 cells

**Fig. 3:**
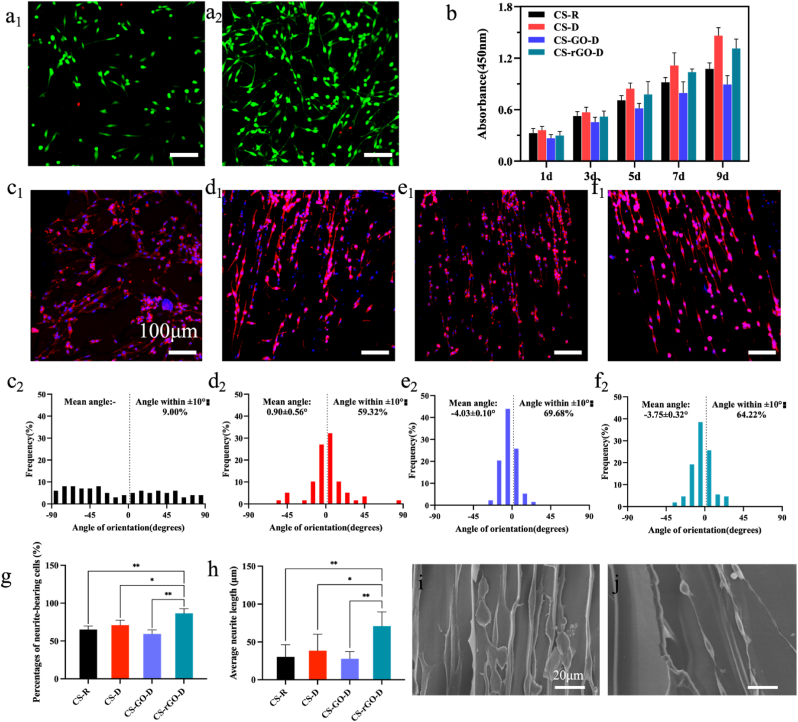
**Characterization of the growth and conditions of PC12 cells cultured in the matrices.** (a_1_, a_2_) Live-dead assay was performed on PC12 cells cultured for 24 h and 48 h of CS-rGO-D matrices. Green and red colors denote live cells and dead cells, respectively. Scale bar: 100 μm. (b) Cell proliferation of PC12 cells seeded on CS-R, CS-D, CS-GO-D, and CS-rGO-D matrices for 1, 3, 5, 7, and 9 days. (c_1_-f_1_) The representative CLSM images of PC12 cells after 7 days of culture on CS-R, CS-D, CS-GO-D, and CS-rGO-D matrices, respectively. PC12 cells were stained with DAPI for nuclei (blue) and rhodamine-phalloidin for F-actin (red). Scale bars: 100 μm. (c_2_-f_2_) Histogram of neurite orientation on corresponding matrices, and 0° represents the direction of the channels. (g, h) Statistical data of the average neurite length and percentages of neurite-bearing cells within different matrices. (i, j) The growth condition of PC12 cells seeded on CS-D and CS-rGO-D matrices under SEM, respectively. Scale bars: 20 μm.

PC12 cells, a nerve model cell type, were initially seeded in CS-R, CS-D, CS-GO-D, and CS-rGO-D matrices and examined for biocompatibility and proliferation. The results indicated that PC12 cells grew well without obvious damage at least 24 and 48 h post-treatment (Fig. 3a_1_, a_2_, S5a-c), which exhibited the good biocompatibility of CS-rGO-D matrices, which might be attributed to the inherent excellent biocompatibility of chitosan, endowing it with the advantage of cell adhesion and proliferation. To further identify the cell proliferation effects of different matrices over a relatively long period, cell proliferation analysis was performed to study the metabolic activity of proliferative PC12 cells inoculated within the matrices for 1, 3, 5, 7, and 9 days. The results demonstrated that the proliferation of PC12 cells was enhanced on CS-rGO-D matrices from day 1 to day 9 (Fig. 3b). Specifically, the number of metabolically active cells grown exhibited good biocompatibility of CS-rGO-D matrices in a long period time. This phenomenon can be explained in two ways: On one hand, the structural orientation with longitudinal and directional extension within the matrices is more inclined to promote PC12 cell growth by providing structural cues. In contrast, the lowest proliferation effect for the CS-GO-D matrix may be induced by the reactive oxygen species created and the absorption of cell nutrition, which causes cell starvation because of the charged functional groups on the GO surface [[45], [46], [47]]. These results support the feasibility of utilizing a directional chitosan-rGO-based composite matrix, CS-rGO-D, as a biocompatible matrix to promote the proliferation of PC12 cells.

Matrices that mimic the structure of natural nerves to induce cell-directed growth benefit nerve development [48]. Thus, the growth status of PC12 cells cultured in different matrices was illustrated (Fig. 3c_1_-f_1_), and it was obvious that PC12 cells adhered, grew, migrated, and neurites extended linearly within the directed multi-channels. To quantitatively analyze neurite alignment, the angle of orientation between the neurites and channels (parallel line defined as 0°) was measured and drawn on day 7 (Fig. 3c_2_-f_2_). Obviously, practically all cells expanded and extended parallel to the channel direction in the directional CS-D, CS-GO-D, and CS-rGO-D matrices, with an absolute value of the mean angle of no more than 4.5°. After fitting the data to a Gaussian distribution, it was showed that 64.22% of the neurites of PC12 cells growing on the CS-rGO-D matrix had an orientation angle within ±10° relative to the channel direction, with a mean angel of −3.75 ± 0.32°. Furthermore, SEM images of the cells on the CS-rGO-D matrix intuitively demonstrated this phenomenon (Fig. 3i and j). In contrast, the neurites of PC12 cells seeded on the CS-R matrix extended in a disorderly manner in all directions, and no Gaussian distribution could be fitted. Additionally, we analyzed several other relative features to disclose the advantageous influence of the CS-rGO-D matrix on cell development on day 7, demonstrated by the maximum average neurite length and the largest percentage of neurite-bearing cells (Fig. 3g and h). As a result, both qualitative and quantitative investigations confirmed that neurites are aligned within longitudinally oriented channels to a large extent. Our results showed that, although in the absence of chemical or biological leading, contact guidance induced from the oriented multi-channels within the matrix was sufficient to provide directional cues for the growth of PC12 cell neurites, bringing them into parallel alignment with the channel direction. Collectively, it has been shown that the CS-rGO-D matrix actively supports the attachment, survival, and directionally oriented growth of PC12 cells in vitro and is viable as a substrate for nerve regeneration.

### 3.3 Influence of CS-rGO-D matrix combined with electrical stimulation in the nerve cell excitability

**Fig. 4:**
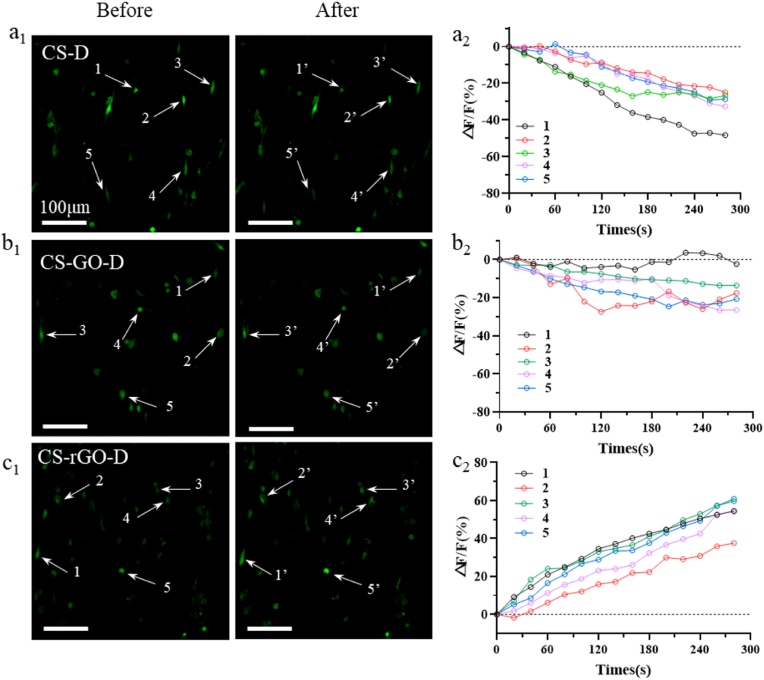
**The fluorescence intensity of intracellular Ca**^**2+**^**under electric stimulation of PC12 cells cultured on different matrices.** (a_1_-c_1_) Intracellular Ca^2+^ imaging (green) of cells under CLSM stained by Fluo-4AM on CS-D, CS-GO-D, and CS-rGO-D matrices, respectively. White arrows indicate the points selected for analysis over the measuring period. Scale bars: 100 μm (a_2_-c_2_) Fluorescence intensity change of Δ*F*/*F*(%) of Ca^2+^ on CS-D, CS-GO-D, and CS-rGO-D matrices, respectively.

It is well-known that the elementary Ca^2+^ events play a major role in controlling neural excitability. For example, Ca^2+^ is pivotal in receiving and transmitting neural signals, as well as in regulating excitability and the changes that underlie synaptic plasticity [49]. Previous reports showed that the voltage pulse stimuli on neural cells from an electrode opened calcium ion channels and increased the calcium ion concentration in cells, resulting in the enhanced fluorescence intensity of Fluo-4-AM dye in the cells [50]. In numerous reports, calcium imaging has been used to characterize PC 12 nerve cell models to evaluate the supporting and guiding effects of substrates on cell growth [51,52]. Therefore, in this study, the improvement in neuron development with functional recovery was measured by detecting the concentration of intracellular calcium ions, Ca^2+^, after the addition of GO or rGO to the matrices. As the influx of Ca^2+^ from the extracellular environment to the intracellular space activates calcium-sensitive proteins, which then mediate neurotransmitter-containing vesicle fusion [53], thus inducing nerve conduction, the difference in electrical stimulation of CS-D, CS-GO-D, and CS-rGO-D matrices on PC12 was analyzed by the intracellular Ca^2+^ fluorescence level of PC12 cells. (Fig. 4a_1_-c_1_). It was obvious that under a series of monophasic cathodic pulses, the fluorescence intensity of PC12 cells showed a downtrend in the CS-D and CS-GO-D matrices, whereas an uptrend appeared in the CS-rGO-D matrix. The relative change in fluorescence intensity Δ*F*/*F* was plotted against stimulation time (Fig. 4a_2_-c_2_), and the cells exhibited a maximum fluorescence intensity increase of approximately 60% under electrical stimuli for the CS-rGO-D matrix. The above results suggest that electrical stimulation can enhance the intracellular Ca^2+^ concentration of nerve cells, and the CS-rGO-D matrix functions as a conductive basis to electrically stimulate the nerve cells and promote nerve signal transmission.

### 3.4 Construction, characterization, and function evaluation of GCr-CSL patch

**Fig. 5:**
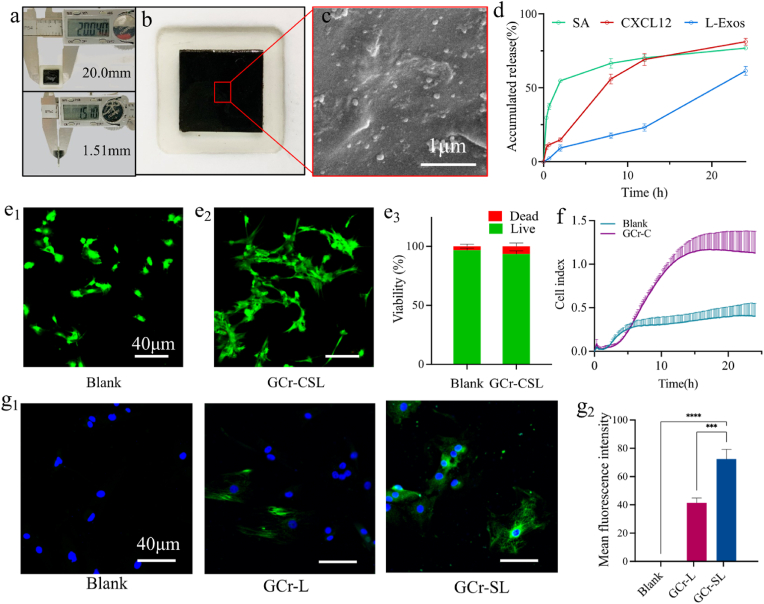
**Schematic diagram of hierarchical structure and characterization of the efficacy of GCr-CSL in recruiting, inhibiting replicative senescence, and promoting nerve differentiation of MSCs *in vitro*.** (a) Size measurement of GCr-CSL patch by vernier caliper. (b) The platform of GCr-CSL in the mold. (c) SEM image of GCr-CSL loaded with L-Exos. Scale bar: 1 μm. (d) The release curves of SA, CXCL12, and L-Exos from the GCr-CSL patch over 24 h (e_1_-e_3_) Live/Dead staining and quantitative analysis of cell viability of MSCs in blank and GCr-CSL patch groups, green color indicates live cells and red color indicates dead cells. (f) Cell index of MSCs recruited by GCr-C over 24 h (g_1_, g_2_) Nestin expression and quantitative analysis in the treated MSCs on Blank, GCr-L, and GCr-SL patches. Scale bar: 40 μm. Statistically significant p-values are indicated as ****p < 0.0001, ***p < 0.001.

We then constructed the CS-rGO based oriented artificial niche, which was fabricated in three steps. First, one slice of the CS-rGO matrix loaded with L-Exos was put into a polydimethylsiloxane square mold, and then the pre-GelMA solution and lithium phenyl-2,4,6-trimethybenzoylphosphinate entrapped with CXCL12 and SA were added to the mold and spin-coated with the CS-rGO matrix. Finally, the whole mold was exposed to UV light to form a patch with a width of 20.04 mm and a thickness of 1.51 mm (Fig. 5a and b). Accordingly, the inner CS-rGO composite matrix entrapping L-Exos was compacted with the outer GelMA hydrogel with encapsulated SA and CXCL12 to form the therapeutic agent co-loaded system, named the GCr-CSL patch. The GelMA hydrogel is biodegradable and relatively thin, so that the excellent mechanical properties of the GCr-CSL patch mainly depend on the CS-rGO-D substrates (Fig. S6d), the biological evaluation of GCr-CSL patch is therefore expected to be intensively explored and verified.

We then evaluated the controlled release of drugs from the GCr-CSL patch. L-Exos vesicles with an average particle size of 174.5 ± 3.0 nm were distributed uniformly within the patch (Fig. 5c, Figs. S6a and b). With the GCr-CSL patch as a reservoir, 55.96% and 81.11% of CXCL12 were released in the first 8 h and the following 24 h, and the respective release percentages for SA were 66.51%, whereas 17.62% and 61.43% of the total proteins incorporated into L-Exos were released in the same period (Fig. 5d). The different release behaviors may be because CXCL12 and SA are dissolved in molecular form and dispersed directly in the matrix, whereas L-Exos need to diffuse through the inner matrix and outer hydrogel successively, and their larger size impedes this process. In addition, the components of L-Exos must pass through the outer membrane themselves, and the relatively longer release time increases the risk of gradual degradation and lowers the maximum release extent (Fig. S6c). These findings support the efficacy of the GCr-CSL patch in releasing CXCL12, SA, and L-Exos in the program, and the programmed release profile corresponds with the dynamic in situ nerve regeneration process in which earlier released CXCL12 recruits MSCs first, and the accompanying SA exerts anti-senescence to maintain the stemness of MSCs for further reproduction and development. The latter L-Exos release finally promotes neuronal differentiation of MSCs that had been recruited and proliferated for some time.

Before determining the effect of the GCr-CSL patch on MSCs' behavior, their biocompatibility was evaluated. Extensive green fluorescence indicated live MSCs and scarce red fluorescence indicated dead MSCs on the GCr-CSL patch. Furthermore, most live cells showed normal morphology under favorable growth conditions, demonstrating negligible cell apoptosis and excellent biocompatibility of the GCr-CSL patch with MSCs (Fig. 5e_1_-e_3_).

The sustained release of CXCL12 resulted in efficient MSC recruitment to the GCr-C patch within 24 h (Fig. 5f). Cellular proliferation is accompanied by obvious cellular senescence, which exerts a detrimental influence on activities such as the proliferative efficiency of cells [54]. We further investigated the senescence status of MSCs in response to SA treatment by detecting the activity of intracellular SA-β-gal, which is expressed in senescent cells and is a widely used biomarker in cellular senescence assays [55]. SA-β-gal levels decreased when treated with SA in a dose-dependent manner, demonstrating the alleviation of cell senescence upon SA treatment (Fig. S7a_1_-a_4_). Furthermore, MSCs on the GCr-S patch showed a significantly lower SA-β-gal level, indicating that entrapment of SA improved the activity of MSCs (Fig. S7b_1_-b_2_). Moreover, it was shown that all L-Exos, GCr-L, and GCr-SL groups could promote neural differentiation of MSCs, as identified by the expression of nestin [56]. However, more than 70% of the MSCs treated with GCr-SL had nestin, which was higher than L-Exos and GCr-L (Fig. 5g_1_, g_2_, Fig. S7c_1_, c_2_), demonstrating that the addition of L-Exos in the patch was effective in driving the differentiation and maturation of functional neuronal lineages from MSCs, and this effect could be enhanced with the assistance of the anti-senescence effect of SA.

### 3.5 GCr-CSL stimulates the recruitment and neural differentiation of MSCs *in vivo*

**Fig. 6:**
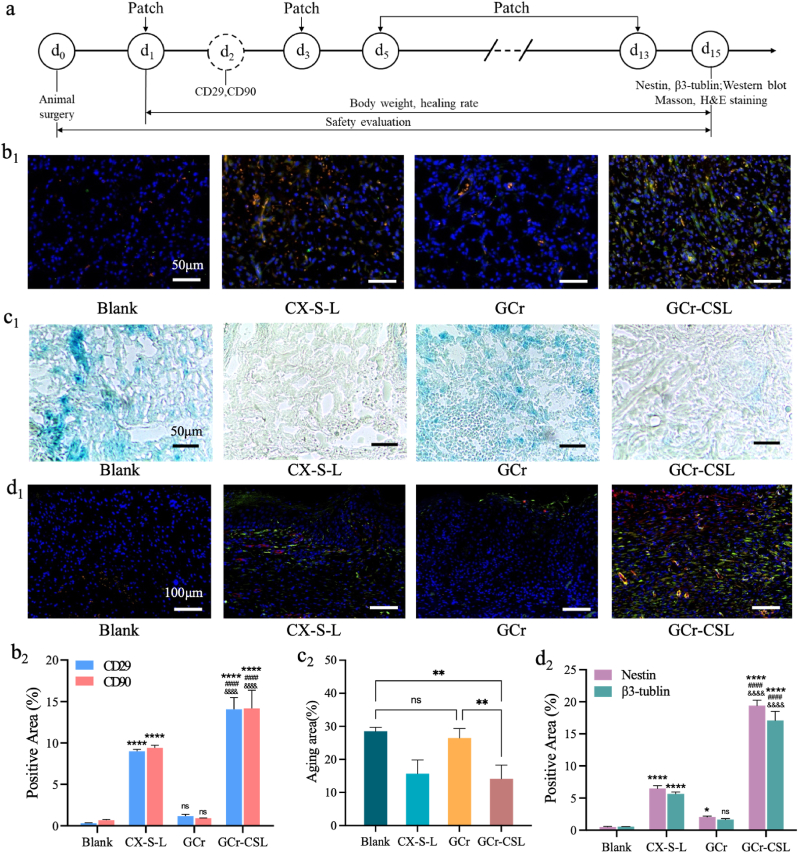
**Investigation of the recruitment, anti-senescence, and neural differentiation of MSCs by GCr-CSL in *vivo.*** (a) The treatment and wound healing assessment scheme was applied to rats. (b_1_,b_2_) The Immunofluorescence staining and quantitative analysis of CD29 and CD90 in the Blank, CX-S-L, GCr, and GCr-CSL groups. Scale bars: 50 μm. (c_1_,c_2_) The expression level and quantitative analysis of SA-β-gal in the Blank, CX-S-L, GCr, and GCr-CSL groups. Scale bars: 50 μm. (d_1_,d_2_) The Immunofluorescence staining and quantitative analysis of nestin and β3-tubulin in the Blank, CX-S-L, GCr, and GCr-CSL groups. Scale bars: 100 μm. Statistical significance is indicated as * p < 0.05, **p < 0.01, ***p < 0.001, **** <0.0001 versus Blank group. #p < 0.05, ##p < 0.05, ###p < 0.001, ####p < 0.0001 versus CX-S-L group. & p < 0.05, && p < 0.01, &&& p < 0.001, &&&& p < 0.0001 versus GCr group.

The full reconstruction of skin with sensory fibers, the ultimate target of wound healing therapy, has been hampered by the complexity of nerve fiber regeneration. Wound therapy therefore aims not only to accelerate healing but also to recover excitation functions through nerve regeneration, for which, GCr-CSL patch is designed for the in situ nerve regeneration. To study the effectiveness of the GCr-CSL patch in recruiting MSCs and promoting nerve regeneration *in vivo*, we established a skin full-excision model in rats in which all the skin and nervous system were removed and the treatment and wound healing-related indicator detection scheme were standardized (Fig. 6a). CD29 and CD90 expression is one of the most consistent phenotypic characteristics of MSCs and is independent of species variation [57]. As shown in Fig. 6b_1_, b_2_, as well as Fig. S8a_1_, a_2_, in the blank, GCr, and GCr-SL groups, scarce CD29 and CD90 expression was identified, indicating that few MSC migrated towards the injury site. In contrast, strong CD29 and CD90 fluorescence intensities were observed in the healed skin of the GCr-CSL, GCr-C, and CX-S-L (drugs distributed in PBS) groups, implying an efficient recruitment effect of CXCL12 on endogenous MSCs. Compared to the CX-S-L group, CD29 and CD90 expression levels in both the GCr-CSL and GCr-C groups increased by approximately 1.5 folds, which might be attributed to the sustained release of CXCL12 from the formulation. These results demonstrated the excellent efficacy of GCr-CSL as a reservoir to release CXCL12, stimulating the in-situ recruitment of MSCs.

It was demonstrated that upon induction of full-thickness wounds, senescent cells become present early in the healing process, around 2–3 days post-wounding, and peak around day six [58]. Therefore, it is of great significance to implement anti-senescence intervention at this stage to accelerate wound repair and improve its repair quality [59]. By detecting the level of SA-β-gal in the wounded skin, it was found that the aging content of cells in the CX-S-L, GCr-SL and GCr-CSL groups was significantly lower than that in the groups without the addition of SA, with the lowest value of 14.15% in the GCr-CSL group (Fig. 6c_1_, c_2_, Fig. S8b_1_, b_2_). To further verify the influence of the combination of L-Exos and SA in the GCr patch on neural differentiation *in vivo*, the expression levels of nestin and β3-tubulin in healed skin were determined. It was found that the highest nestin and β3-tubulin expression was identified in the healed skins of the GCr-CSL group on day 15 post-treatment (Fig. 6d_1_, d_2_, Fig. S8c_1_, c_2_). This *in vivo* nerve regeneration effect of GCr-CSL was confirmed by western blotting (Fig. S8d_1_). The expression levels of nestin in the GCr-CSL group were 1.79-, 1.26-, and 1.56-fold higher than those in the CX-S-L, GCr-C, and GCr-SL groups, respectively. The corresponding increase for β3-tubulin was 2.16 folds, 1.39 folds, and 1.70 folds (Fig. S8d_2_) than that of blank control.

Interestingly, considering the results of immunofluorescence imaging and western blotting, we found that the degree of neural differentiation increased in the order of CX-S-L, GCr-SL, and GCr-C groups, indicating that the most important precondition for in situ nerve regeneration was the effective recruitment of MSCs towards the injury site. The combination of SA and L-Exos also could promote nerve regeneration to some extent, based on the epibiotic MSCs in the surrounding tissue around the wound edge.

The above findings demonstrate that the collaboration of CXCL12, SA, and L-Exos endows the GCr-CSL patch with the most capacity for nerve regeneration *in vivo* by releasing the active ingredients in a sustained manner, which plays multiple roles in recruiting MSCs, performing anti-senescence functions, and promoting neural differentiation.

### 3.6 GCr-CSL promotes wound healing with high biosafety

There is growing evidence that the skin has constant and complex interactions with the nervous, and the cutaneous innervation is an important modulator of the normal wound healing process. The effects of nerve regeneration on wound healing include but are not limited to the following aspects: First, increase the blood supply to the tissue around the wound by initiating the neurogenic inflammatory response and neurotrophic action [60]. Second, promote the proliferation of fibroblasts and keratinocytes and increase the wound contraction [61]. Third. Promote skin wound healing by interacting with the immune system and releasing neuropeptides [60]. Therefore, the in situ nerve regeneration strategy based on endogenous stem cells shows considerable potential in improving the speed and quality of wound healing and skin tissue regeneration.

**Fig. 7:**
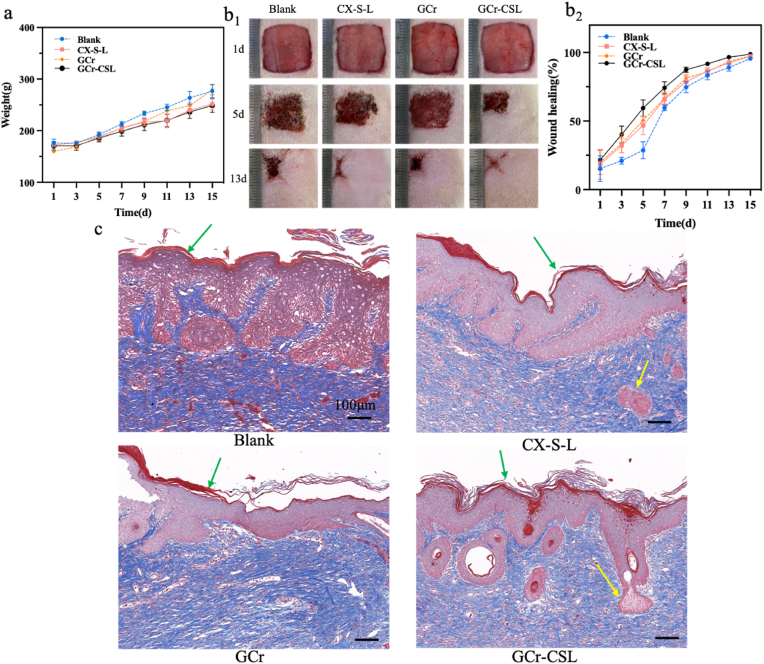
**Evaluation of GCr-CSL patch in promoting wound healing.** (a) Body weights of rats at different time points. (b_1_, b_2_) Wound healing status and rates of rats at different time points. (c) Images of Masson's trichrome staining of healed skin on day 15 post-treatment. Scale bars:100 μm. The green arrows indicate the epidermis and the yellow arrows indicate the skin appendages.

We investigated the influence of GCr-CSL on wound healing by comparing the closure rate and histology of wounded skin after different treatments. The body weights of all the tested rats increased throughout the experimental period, and there was no significant difference between the groups. (Fig. 7a, Fig. S9a). When compared with other groups, the GCr-CSL group showed the highest acceleration in wound healing, and this phenomenon was observed only on day 3 and persisted throughout the healing course (Fig. 7b_1_, b_2_, Fig. S9b_1_, b_2_). Skin appendages and nerves are both important constituents for fully functional skin. Masson's trichrome staining was therefore used to examine the histological properties of the healed skin on day 15 post-treatment. The healed skin in the blank, GCr, and GCr-C groups displayed a wide area of dense dermis devoid of skin appendages, with scar characteristics (Fig. 7c, Fig. S9c). In comparison, appendage-like structures were found in the healed skin of the GCr-SL, CX-S-L, and GCr-CSL groups (indicated by yellow arrows). In addition, the highest density of skin appendage-like structures was observed in the GCr-CSL group, and it was demonstrated that the skin collagen in the GCr-CSL group was organized in a three-dimensional basket-weave-like network similar to that of normal skin [62]. Thus, the GCr-CSL patch was effective in accelerating wound healing and inhibiting scar formation, which may be due to the paracrine function of recruited MSCs [63]. The main organs of the tested rats showed no pathological changes after hematoxylin-eosin staining (Fig. S10). These results demonstrate the good biocompatibility and biosafety of GCr-CSL for *in vivo* bioapplications.

## 4 Discussions

In situ nerve regeneration is a complex process regulated by a variety of physical and biological factors and therefore requires treatment by multiple signals based on collaborating effects. As for physical factors, mechanical strength adapted to the deformation of damaged tissue, a suitable biodegradation rate satisfying the tissue regeneration speed, and surface amphiphilicity for cell adhesion and growth are basic requirements to ensure wound healing. In addition, effective wound healing needs to be accompanied by sensory recovery, presupposing the regeneration of neurons and the reconstruction of the conduction system, which has a significant relationship with the aligned multi-porous morphology and the enhancement of bioelectrical transmission. In addition to biophysical cues, biochemical cues also need to be considered, such as chemoattractants to recruit MSCs, natural bioactive small molecules to anti-senescence, and plant-derived exosomes to promote neural differentiation. Therefore, it is essential to construct an artificial biomimetic niche to build a microenvironment that provides both biophysical and biochemical external interventions for cell mobilization, proliferation, and differentiation to maximize wound healing with nerve regeneration. However, few wound healing strategies have focused on combining biophysical and biochemical cues, resulting in limited effects.

Chitosan is a well-known neural scaffold matrix material due to its positive properties such as biocompatibility, biodegradability, and nontoxicity, however, the low mechanical strength under physiological conditions of chitosan matrices has initially limited their use as nerve guidance conduits [64]. At present, surface modification, blending with other matrix materials and advanced manufacturing processes are usually used to prepare chitosan matrix with appropriate mechanical properties, porosity and degradation rate [65]. However, the preparation of current common material systems is complicated and relatively high cost, which may damage the characteristics of chitosan matrix itself, and some additives have potential toxic and side effects [66]. To address those problems, graphene derivatives, such as reduced graphene oxide (rGO) with a high specific surface area, mechanical strength, and conductivity can be co-manufactured to remedy the above deficiency and improve performance. Compared with the current widely used additive fillers, graphene materials have obvious advantages, which can improve various defect characteristics of chitosan scaffolds at a very low additive amount [67]. They have been confirmed to play a positive role in the growth, differentiation, and development of nerve pedigree cells through the electrical stimulation of nerve cells [68,69]. In addition, the adjustable surface properties and manufacturing process of graphene-based derivatives such as GO and rGO make them suitable for the fabrication of commodifiers in combination with other materials and neuron-like structures to align the arrangement of neurons [70]. Therefore, in this work, a self-assembled matrix based on chitosan and rGO crosslinking that coordinate the mechanical properties, amphipathy, and conductivity of materials for tissue engineering was put forward.

Subsequently, with the photo-crosslinking GelMA dressing compacted as the outer layer, the matrix-gel hybrid system was further incorporated with L-Exos into the inner matrix for nerve differentiation and CXCL12 and SA into the outer gel for MSC recruitment and anti-senescence, respectively, to form the GCr-CSL patch. As expected, the diverse active ingredients could be released in a programmed file, which is satisfied with the in situ nerve regeneration process, recruiting MSCs first, preventing the senescence of MSCs subsequently during proliferation, and promoting neural differentiation. Which not only provides a matrix guiding and orienting nerve regrowth but also creates an interface that allows multiple biological factors to interact with cells, facilitating nerve structural and functional recovery. The application of the GCr-CSL patch *in vivo* significantly promoted MSC differentiation into neurons and wound healing with skin appendage regeneration due to the cooperative action of the therapeutic exoskeleton, natural anti-aging agent, recruitment factor, and plant-derived exosomes. To sum up, in this study, we successfully developed a novel multi-functional matrix-gel hybrid platform, the GCr-CSL patch, which integrates the dual transmission of biophysical and biochemical signals to promote wound healing with skin nerve regeneration.

In addition, in our work, compared with the common manufacturing, the orientation freezing technique has obvious advantages [71]. By changing the structure of the template, the shape of the reactor and the properties of the dispersing medium, various matrices with different microstructure and ordered structure can be obtained [72]. The CS-rGO-D matrix was prepared as the inner layer using the freeze-casting method, which has the unique advantage of providing a linearly aligned porous structure for the guidance and directional induction of regenerating nerves in the inner microenvironment, and also has the characteristics of mechanical robustness, biocompatibility, and biodegradability, which is preferable as a tissue substitute to facilitate tissue regeneration. Furthermore, the matrix prepared by orientation freezing technique is easy to obtain, customizable, low cost, and does not introduce new potentially toxic substances, such as crosslinkers, which shows that CS-rGO-D has the prospect of large-scale manufacturing. This fabrication strategy provides new insights into the preparation of other functional, inexpensive, and effective tissue graft substitutes for medical applications.

When evaluating further applications, there are still some limitations. Although this work has achieved satisfactory results in promoting nerve regeneration in rats, there are still some problems and challenges to be solved. For example, a variety of biological therapeutic agents and physical factors mentioned in this work are determined to be useful in nerve regeneration, but how to coordinate the combination of multiple factors to achieve the best therapeutic effect still remains to be explored. Therefore, how to make the patch preparation quality stable and controllable is the key to its clinical application and industrial production. In addition, the molecular mechanism of this patch therapy is still unclear and needs more genomic analysis and molecular level inquiry to clarify. Given that the patch is biodegradable at the wound site, easy to clean up, does not cause residue, does not cause visceral damage and has extremely high biosafety and biocompatibility for *in vivo* bioapplications. After establishing a systematic quality analysis index system, we have confidence and expectation for the clinical application of the patch.

## 5 Conclusions

In summary, the oriented freezing technology was used to assemble chitosan and graphene into aerogels with longitudinally ordered channels and serve as nerve scaffolds, which can effectively induce the transmission of nerve signals and promote the directional growth and differentiation of MSCs. It was combined with L-Exos and GelMA loaded with CXCL12 and Salvianolic acid A to form GCr-CSL patch. The GCr-CSL patch shows a remarkable speed in regenerating tissues through the controlled release of diverse therapeutics with the enhancement of bioelectricity signal transmission. This multifunctional patch highlights the prospect of combining biophysical and biochemical factors to build an adaptive platform and serves as a reference for integrated therapy for regenerative medicine.

## Credit author statement

**Lihua Peng**: Conceptualization, Writing, review and editing, Resources, Supervision, Funding acquisition, Project administration. **Aiping Liu**: Conceptualization, review and editing. **Minhong Tan**: Investigation, Data curation, Writing, Editing. **Weizhong Xu**: Investigation, Data curation, Writing, Editing. **Ge Yan**: Investigation, Data curation. **Yang Xu**: Investigation, Data curation. **Qiyao Xiao**: Investigation, Data curation.

## Ethics approval and consent to participate

All animal experimental procedures were performed in obedience to the guidelines and protocols of the Animal Experimental Ethics Committee of Zhejiang University (ZJU20170733).

## Declaration of competing interest

The authors declare that they have no known competing financial interests or personal relationships that could have appeared to influence the work reported in this paper.

## Data Availability

Data will be made available on request.

## Abbreviations

GelMA: Methacrylate gelatin
MSC: Mesenchymal stem cell
BMSC: Bone marrow mesenchymal stem cell
CS: Chitosan
GO: Graphene oxide
rGO: Reduced graphene oxide
CXCL12: Chemokine C-X-C motif chemokine ligand 12
SA: Salvianolic acid A
ELVs: Exosome-like vesicles
L-Exos: Ligusticum chuanxiong hort
GCr-CSL: GelMA-coated CXCL12, SA, and L-Exos-loaded CS-rGO
