# Supplementary material for: Oriented artificial niche provides physical-biochemical stimulations for rapid nerve regeneration
Source: Mater Today Bio. 2023 Jul 20;22:100736. doi: 10.1016/j.mtbio.2023.100736 (PMC10374615; doi:10.1016/j.mtbio.2023.100736)
Supplement: Supplementary file 1 [file mmc1.docx]

Supporting Information for

**Oriented** **artificial niche** **provides physical-biochemical stimulations for rapid nerve regeneration**

*Minhong Tan^1,4#^, Weizhong Xu^3#^, Ge Yan^1^, Yang Xu^1^, Qiyao Xiao^1^, Aiping Liu^3,^*, Lihua Peng^1,2,^**

^1^ College of Pharmaceutical Sciences, Zhejiang University, Hangzhou 310058, PR China

^2^ State Key Laboratory of Quality Research in Chinese Medicine, Macau University of Science and Technology, Macau, PR China

^3^ Key Laboratory of Optical Field Manipulation of Zhejiang Province, Zhejiang Sci-Tech University, PR China

^4^ College of Materials Science and Engineering, Zhejiang University, Hangzhou 310027, PR China

^#^These authors contributed equally to this work.

*Corresponding authors.

Lihua Peng, Ph.D, Associate Professor.

College of Pharmaceutical Sciences, Zhejiang University, 866# Yuhangtang Road, Hangzhou, 310058, P.R. China. Email: [lhpeng@zju.edu.cn](mailto:lhpeng@zju.edu.cn) Tel/Fax: +86-571-88981231

Aiping Liu, Ph.D, Professor.

Key Laboratory of Optical Field Manipulation of Zhejiang Province, Zhejiang Sci-Tech University, 928# Street 2, Hangzhou, 310018, P. R. China. Email: [liuaiping1979@gmail.com](mailto:liuaiping1979@gmail.com) Tel/Fax: +86-571-86843468

**Tables S1.** The density, porosity and compression modulus (dry and wet environment) of the various nerve matrices.

| Scaffolds | Density (mg/cm^3^) | Porosity (%) | Compression modulus (kPa) | |
| --- | --- | --- | --- | --- |
|  |  |  | Dry | Wet |
| CS-R | 81.43±2.08 | 88.72±2.53 | 5.07±5.47 | 2.16±6.27 |
| CS-D | 82.07±2.51 | 90.23±3.81 | 122.53±3.44 | 28.95±3.94 |
| CS-GO-D | 74.80±3.99 | 91.63±2.62 | 156.55±3.59 | 43.00±5.66 |
| CS-rGO-D | 70.34±5.53 | 92.58±2.69 | 149.83±2.22 | 37.28±8.57 |

**
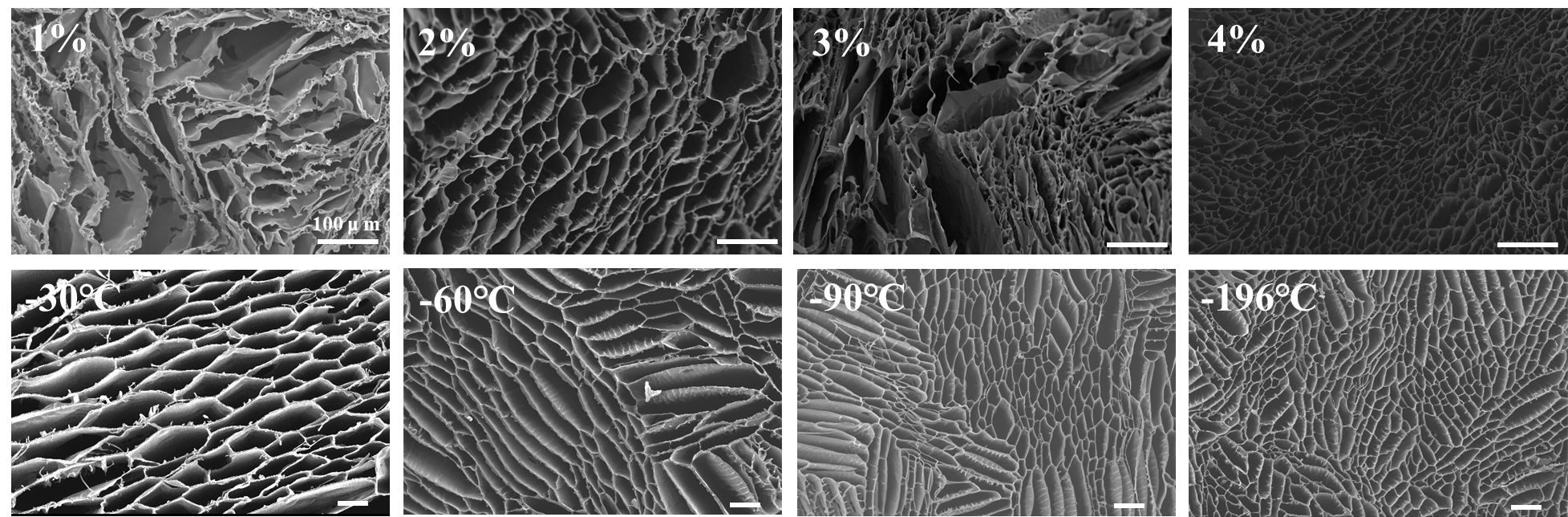
**

**Figure S1. Pore morphology of CS matrices at different concentrations and different freezing temperatures.** Scale bars: 100 μm.

**Figure S2. The microstructure of neural matrices after crosslinking.** Cross section diagram (a_1_-c_1_) and longitudinal section diagram (a_2_-c_2_) of CS-R, CS-D and CS-GO-D, respectively. Scale bars: 200 μm. (d) Pore sizes of CS-R, CS-D, and CS-GO-D matrices. respectively.

**
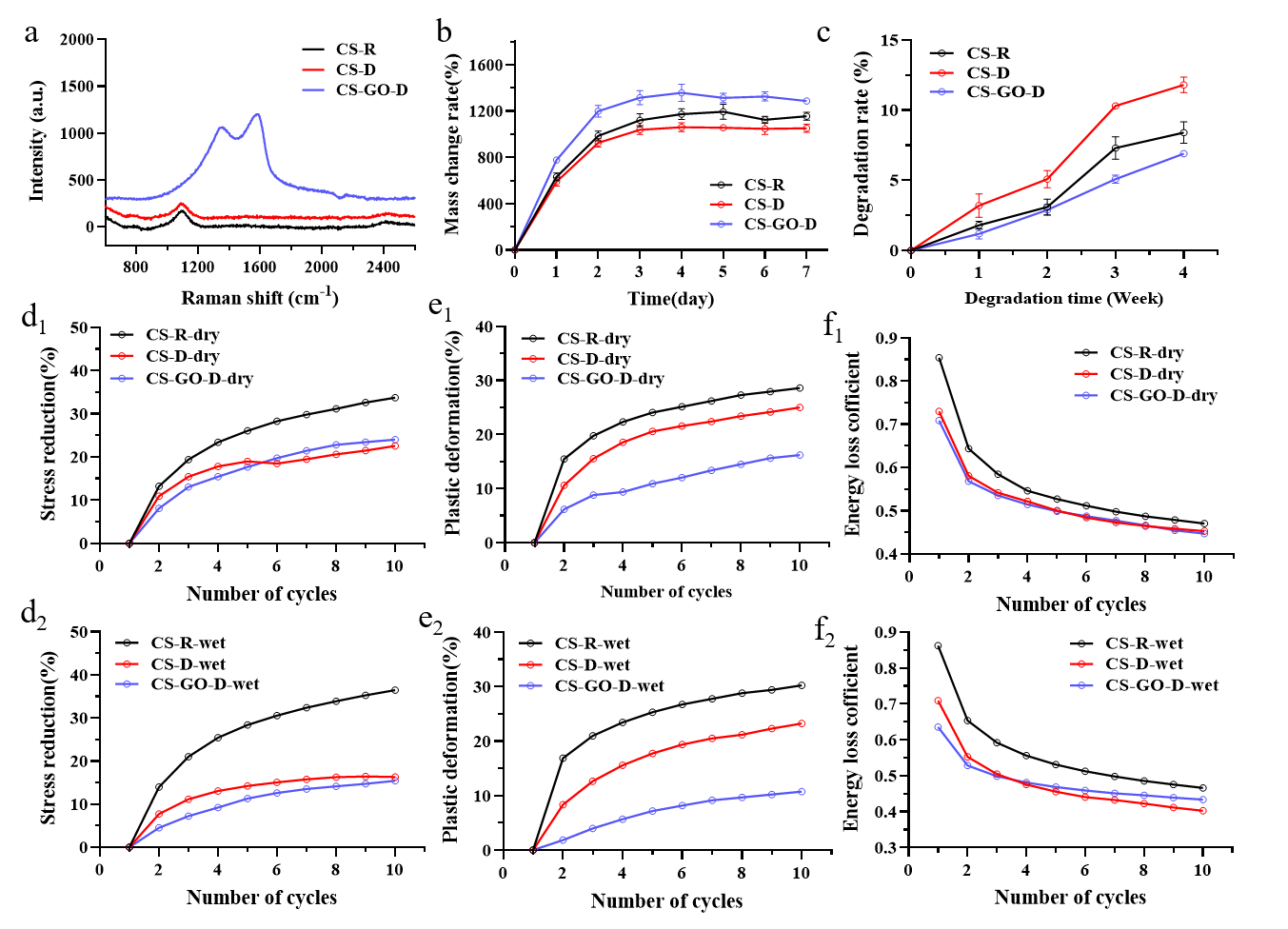
**

**Figure S3. The identification and characterization of CS-R, CS-D and CS-GO-D matrices.** (a) Raman spectrum, (b) Swelling ratio, (c) Degradation rate of CS-R, CS-D and CS-GO-D matrices in dry/wet environment, respectively. Stress reduction, plastic deformation, energy loss coefficient of CS-R, CS-D and CS-GO-D matrices under dry environment (d_1_-f_1_) and wet environment (d_2_-f_2_), respectively.

**Figure S4.** Stress-strain curve of CS-R (a_1_, a_2_), CS-D (b_1_, b_2_), CS-GO-D (c_1_,c_2_) and CS-rGO-D (d_1_, d_2_) matrices under dry environment and wet environment, respectively.


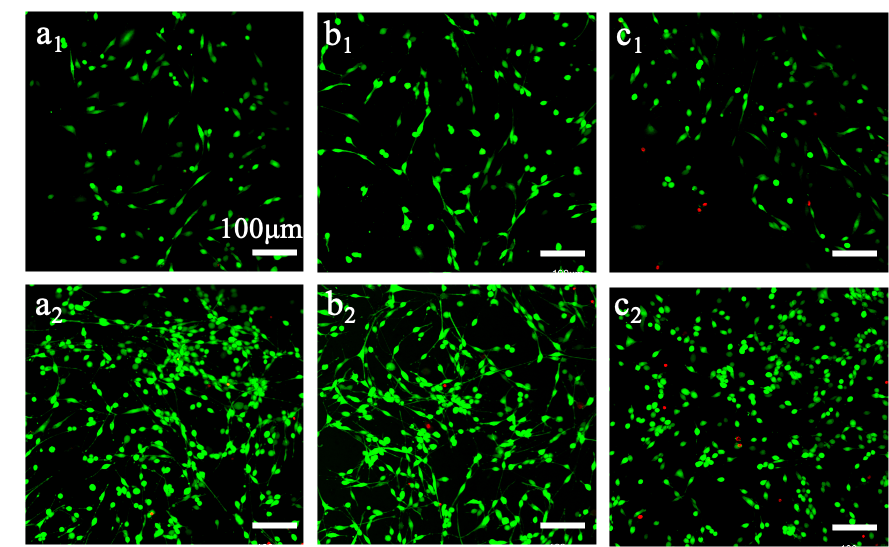


**Figure S5: Biocompatibility of matrices.** Live-dead assay performed on PC12 cells cultured for (a_1_-c_1_) 24h, and (a_2_-c_2_) 48h of (a_1_, a_2_) CS-R, (b_1_, b_2_) CS-D, and (c_1_, c_2_) CS-GO-D matrices. Green and red color denote live cells and dead cells, respectively. Scale bar: 100 μm.

**Figure S6**. (a) TEM image of L-Exos. Scale bars: 200 nm. (b) Size distribution and Zeta potential of L-Exos. (c) Degradation of GCr-CSL. (d) Stress-strain curve of GCr-CSL patch.

**Figure S7.** (a_1_-a_4_) SA-β-gal staining and cell aging rate of MSCs at different SA concentration. Scale bar: 40 μm. (b_1_, b_2_) Anti-senescence effect of GCr-S on MSCs. Scale bar: 40 μm. (c_1_,c_2_) Neatin expression level of MSCs treated in blank and L-Exos. Blue color indicates cell nucleus stained by DAPI. Green color indicates nestin. Scale bar: 40 μm. Statistical significance is indicated as * p < 0.05, ** p < 0.01, *** p < 0.001, **** ＜0.0001 versus Blank group.


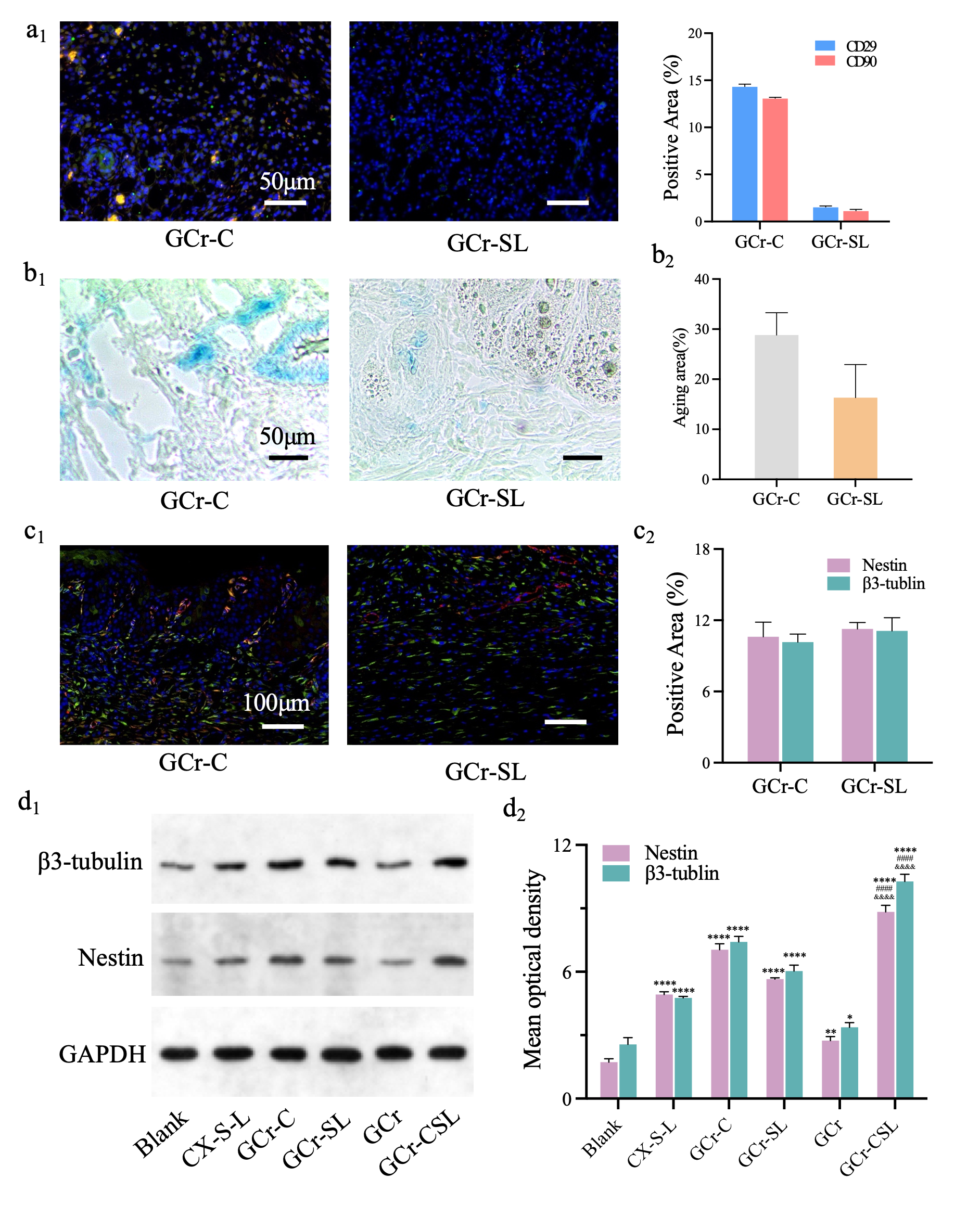


**Figure S8.** (a_1_,a_2_) The expression level of CD29 and CD90 in the GCr-C and GCr-SL groups. Scale bar: 50 μm. (b_1_, b_2_) The expression level and quantitative analysis of SA-β-gal in the GCr-C and GCr-SL groups. Scale bar: 50 μm. (c_1_,c_2_) The immunofluorescence staining expression level of nestin and β3-tubulin in the GCr-C and GCr-SL groups. (d_1_, d_2_) The expression levels and quantitative analysis of nestin and β3-tubulin in different groups. Scale bar: 100 μm. Statistical significance is indicated as * p < 0.05, ** p < 0.01, *** p < 0.001, **** ＜0.0001 versus Blank group. # p < 0.05, ## p < 0.05, ### p < 0.001, #### p ＜0.0001 versus CX-S-L group. & p < 0.05, && p < 0.01, &&& p < 0.001, &&&& p ＜0.0001 versus GCr group.

**
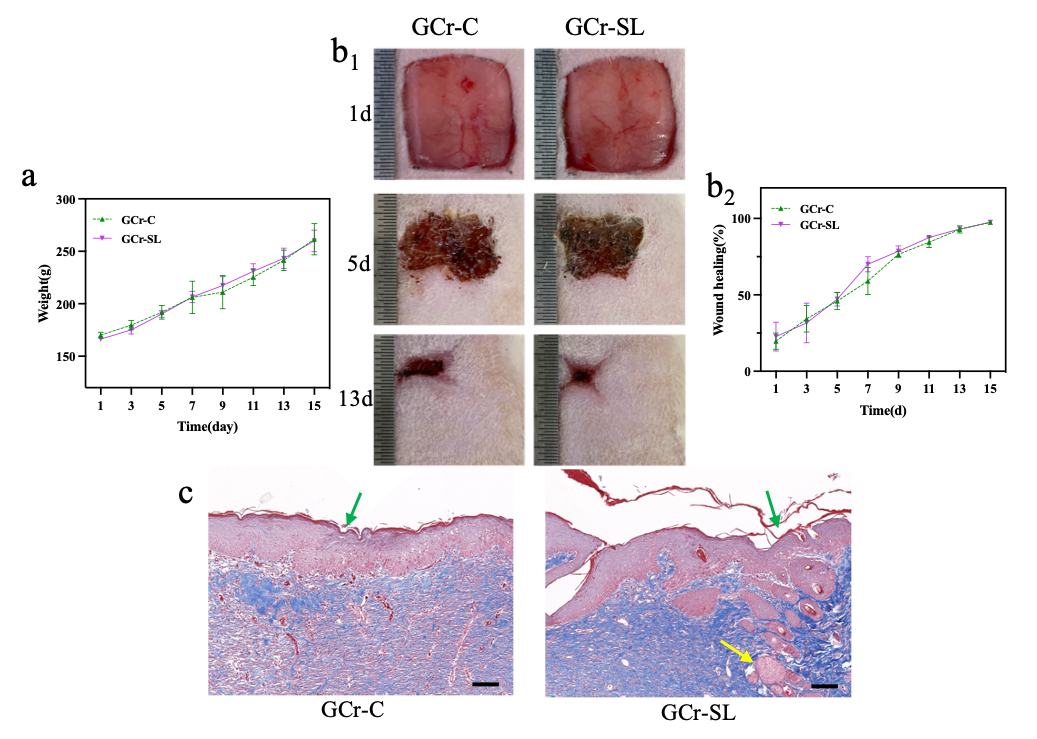
**

**Figure S9.** (a) Body weights of GCr-C and GCr-SL groups at different time points. (b_1_, b_2_)Wound healing status and rates of GCr-C and GCr-SL groups for 13 days. (c) Images of Masson's trichrome staining of healed skin on day 15 post-treatment. Scale bars:100 μm. The green arrows indicate the epidermis and the yellow arrows indicate the skin appendages.


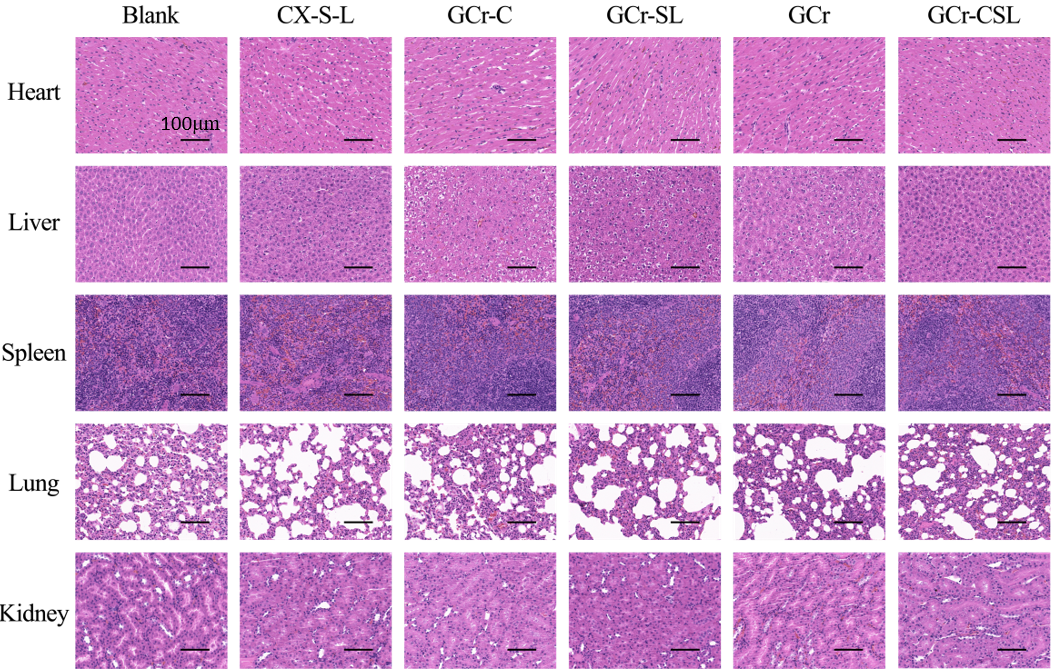


**Figure S10.** Hematoxylin-eosin staining of heart, liver, spleen, lung and kidney in blank,

CX-S-L, GCr-C, GCr-SL, GCr and GCr-CSL groups.
